# High serum concentration of vitamin D may protect against multiple sclerosis

**DOI:** 10.1177/2055217319892291

**Published:** 2019-12-06

**Authors:** Martin Biström, Lucia Alonso-Magdalena, Oluf Andersen, Daniel Jons, Martin Gunnarsson, Magnus Vrethem, Johan Hultdin, Peter Sundström

**Affiliations:** Department of Pharmacology and Clinical Neuroscience, Umeå University, Sweden; Department of Neurology, Skåne University Hospital in Malmö/Lund and Institution of Clinical Sciences, Neurology, Lund University, Sweden; Department of Clinical Neuroscience, Institute of Neuroscience and Physiology, the Sahlgrenska Academy, University of Gothenburg, Sweden; Department of Neurology, Faculty of Medicine and Health, Örebro University, Sweden; Department of Neurology and Department of Clinical and Experimental Medicine, Linköping University, Sweden; Department of Medical Biosciences, Clinical Chemistry, Umeå University, Sweden; Department of Pharmacology and Clinical Neuroscience, Umeå University, Sweden

**Keywords:** Vitamin D, multiple sclerosis, case–control studies, risk factors, epidemiology, 25-hydroxyvitamin D

## Abstract

**Background:**

High 25-hydroxyvitamin D concentrations have been associated with a reduced risk of multiple sclerosis, with indications of a stronger effect among young individuals.

**Objective:**

Investigate the 25-hydroxyvitamin D association with multiple sclerosis and test if this association is age dependent.

**Methods:**

Prospectively drawn blood samples from individuals later developing relapsing–remitting multiple sclerosis and controls matched for biobank, sex, age and date of sampling, were analysed with liquid chromatography tandem mass spectrometry.

**Results:**

High levels of 25-hydroxyvitamin D (top quintile) were associated with a reduced multiple sclerosis risk (odds ratio 0.68, 95% confidence interval 0.50–0.93).

**Conclusion:**

These findings further support a role for vitamin D in MS aetiology.

## Introduction

Higher serum concentrations of 25-hydroxyvitamin D (25(OH)D) have repeatedly been associated with a decreased risk of multiple sclerosis (MS) development in nested case–control studies^1–3^ with one study showing a larger effect before 20 years of age.^1^ Additional support for a causal role of vitamin D in MS aetiopathogenesis comes from Mendelian randomisation studies^4,5^ but this subject remains controversial.^6^

In this study we aimed to test the hypothesis that high 25(OH)D concentrations reduce the risk of developing MS, with a more pronounced effect among young individuals, by comparing blood samples from healthy controls to samples from individuals who later developed relapsing–remitting multiple sclerosis (RRMS). To achieve these goals, we accessed six Swedish biobanks specifically chosen because they include plasma or serum drawn at a young age.

## Materials and methods

### Case ascertainment

In this nested case–control study we accessed five Swedish microbiological biobanks associated with university hospitals in Umeå, Örebro, Göteborg, Skåne and Linköping and one biobank from the Public Health Agency of Sweden (PHAS), to obtain serum or plasma from a total of 670 individuals who later developed RRMS and 670 controls matched for biobank and sex, and with decreasing priority for date of sampling and age. These biobanks contain remainders from serological analysis in routine clinical practice. Cases were identified either through crosslinking with the Swedish MS registry (www.neuroreg.se) or a local MS/possible MS database, and a total of 665 complete sets of cases and controls were included in the final analysis (see Supplementary Figure 1). All samples from MS patients were collected 8 years prior to symptom onset (in median) and all participants were below 40 years of age at the time of sampling. The absolute mean difference between cases and controls was 6 days for the date of sampling and 152 days for the age at sampling.

### Laboratory analysis

The concentration of 25(OH)D_3_ was analysed using liquid chromatography tandem mass spectrometry (LC-MS/MS) as described previously.^7^ Samples for matched cases and controls were analysed immediately after each other and in random order, and technicians were blinded to case–control status.

### Statistical analysis

We modelled 25(OH)D_3_ levels as quintiles, derived from the distribution among controls, separately for each biobank as the levels differed significantly between them (Kruskal–Wallis test *P* ≤ 0.001). These quintile assignments were used in a pooled analysis that included all individuals. The Mann–Whitney U-test was used to test differences between groups, odds ratios (ORs) and *P* for trend over quintiles were calculated using conditional logistic regression. Age was stratified into three groups based on the age at sample draw, less than 20, 20–29 and 30–39 years of age. If a case–control set was on different sides of an age cut-off they were assigned to either the youngest or oldest group containing either a case or control, in order to increase power in the smaller groups. IBM SPSS statistics version 23 (IBM Corporation, New York, NY, USA) was used for statistical analysis.

### Ethical considerations

This study was approved by a local regional ethical review board in Umeå (2011-198-31M). No written informed consent was required for participation.

## Results

Median 25(OH)D_3_ did not differ between cases and controls (Table 1). Being in the top 25(OH)D_3_ quintile was significantly associated with a decreased risk of MS in the total cohort (OR 0.68, 95% confidence interval (CI) 0.50–0.93) (Table 2). A sensitivity analysis excluding the PHAS biobank, which had higher levels compared to the others, yielded an OR of 0.69 (95% CI 0.49–0.97) when using the median cut-off for the remaining biobanks (72 nmol/L). Subgroup analyses in different age strata were not significant and we found no trend over 25(OH)D_3_ quintiles.

**Table 1.** Characteristics of cases and controls.

	*n*	Cases	*n*	Controls	*P* value
Sex (M/F)	665	16.2/83.8%	665	16.2/83.8%	
Age at sampling, years	665	25 (21–29)	665	25 (21–29)	
Age at disease onset, years	665	33 (28–40)	n.a.		
Time from sampling until disease onset, years	665	8 (4–13)	n.a.		
Biobank – latitude					
Umeå – 63°N	102	15.3%	102	15.3%	
Vitamin D3		47 (37–61)		53 (39–68)	0.07
Samples collected between, years		1976–2007		1976–2007	
PHAS – n.a.	137	20.6%	137	20.6%	
Vitamin D3		59 (43–75)		56 (39–77)	0.64
Samples collected between, years		1972–2001		1972–2001	
Örebro – 59°N	29	4.3%	29	4.3%	
Vitamin D3		52 (41–63)		50 (36–70)	0.96
Samples collected between, years		1994–2008		1994–2008	
Göteborg – 57°N	47	7.1%	47	7.1%	
Vitamin D3		56 (41–65)		55 (44–67)	0.97
Samples collected between, years		1995–2009		1995–2009	
Skåne – 55°N	311	46.8%	311	46.8%	
Vitamin D3		52 (41–68)		52 (40–70)	0.93
Samples collected between, years		1977–2007		1977–2007	
Linköping – 58°N	39	5.9%	39	5.9%	
Vitamin D3		43 (30–57)		51 (32–61)	0.33
Samples collected between, years		1993–2009		1993–2009	
All subjects	665		665		
Vitamin D3		53 (40–67)		53 (39–70)	0.50
Age group <20 years	142		142		
Vitamin D3		49 (38–64)		51 (39–67)	0.47
Age group 20–29 years	374		374		
Vitamin D3		53 (41–67)		53 (39–71)	0.73
Age group 30–39 years	149		149		
Vitamin D3		55 (41–72)		56 (42–73)	0.83

PHAS: Public Health Agency of Sweden.

Median (25th–75th percentiles) for continuous variables and percentages for proportions.

Vitamin D concentrations expressed as nmol/L.

**Table 2.** Associations of vitamin D_3_ concentration and MS stratified by biobank and age.

	Vitamin D categories	Cut-off nmol/L	Number of (%)		OR	95% CI
			Cases	Controls		
Biobank
Umeå	Quintile 1–4	38, 48, 59	90 (88.2)	81 (79.4)	ref	
	Quintile 5	≥73	12 (11.8)	21 (20.6)	0.47	0.20–1.1
PHAS	Quintile 1–4	37, 50, 63	114 (83.2)	109 (79.6)	ref	
	Quintile 5	≥82	23 (16.8)	28 (20.4)	0.75	0.38–1.5
Örebro	Quintile 1–4	35, 48, 60	26 (89.7)	23 (79.3)	ref	
	Quintile 5	≥72	3 (10.3)	6 (20.7)	0.40	0.08–2.1
Göteborg	Quintile 1–4	41, 50, 59	41 (87.2)	37 (78.7)	ref	
	Quintile 5	≥71	6 (12.8)	10 (21.3)	0.43	0.11–1.7
Skåne	Quintile 1–4	38, 47, 58	252 (81.0)	248 (79.7)	ref	
	Quintile 5	≥73	59 (19.0)	63 (20.3)	0.90	0.58–1.4
Linköping	Quintile 1–4	27, 42, 55	37 (94.9)	31 (79.5)	ref	
	Quintile 5	≥70	2 (5.1)	8 (20.5)	0.25	0.05–1.2
All	Quintile 1–4		560 (84.2)	529 (79.5)	ref	
	Quintile 5		105 (15.8)	136 (20.5)	0.68	0.50–0.93
All^a^	Quintile 1		134 (20.1)	133 (20.0)	ref	
	Quintile 2		142 (21.4)	133 (20.0)	1.1	0.77–1.5
	Quintile 3		131 (19.7)	130 (19.5)	0.99	0.71–1.4
	Quintile 4		153 (23.0)	133 (20.0)	1.1	0.78–1.6
	Quintile 5		105 (15.8)	136 (20.5)	0.72	0.49–1.1
Age group
<20	Quintile 1–4		126 (88.7)	118 (83.1)	ref	
	Quintile 5		16 (11.3)	24 (16.9)	0.60	0.29–1.2
20–29	Quintile 1–4		313 (83.7)	297 (79.4)	ref	
	Quintile 5		61 (16.3)	77 (20.6)	0.70	0.46–1.1
30–39	Quintile 1–4		121 (81.2)	114 (76.5)	ref	
	Quintile 5		28 (18.8)	35 (23.5)	0.72	0.39–1.3

MS: multiple sclerosis; OR: odds ratio; CI: confidence interval; PHAS: Public Health Agency of Sweden.

^a^In the total cohort, *P* for trend over quintiles was 0.24.

## Discussion

Although cases and controls did not significantly differ in median levels of 25(OH)D and there was no significant trend over quintiles, we did find a decreased MS risk among individuals with concentrations in the top quintile. These findings suggest that there may exist a threshold located within the higher range of 25(OH)D levels (cut-off 70–82 nmol/L in the six biobanks) above which the effect of 25(OH)D modulates MS risk. This is in line with the findings of one earlier study using 75 nmol/L as a cut-off.^2^ Data from the currently largest pre-symptomatic study, performed in a Finnish maternity cohort,^3^ seem to indicate that seasonally corrected levels above 50 nmol/L are protective when compared to less than 30 nmol/L. In that study, 6% of cases and 7.5% of controls were above 50 nmol/L, compared to 54.6% and 56.1% in our study. Although we found higher vitamin D levels, they are in line with previously published population-based studies in our region.^8^ Differences in methodology, including 25(OH)D assay and the use of seasonal correction of multiple samples from each individual in the Finnish study, may explain some of the differences in absolute 25(OH)D and a direct comparison between the studies may therefore be inappropriate.^9^

A strength of our study is the relatively large number of individuals below 20 years of age, enabling comparisons of different age strata. These analyses did not yield any significant findings, however, but the effect sizes converge with earlier studies.^1,2^ Furthermore, this is to our knowledge the first study applying the gold standard method LC-MS/MS.

The main limitation in our study is that the samples came from six unique biobanks, with different pre-analytical procedures and geographically distinct catchment areas, both of which may influence the results as well as provide a geographical explanation of why serum concentrations of vitamin D differed between biobanks. To minimise this, we matched cases and controls from the same biobank and defined quintiles separately for each biobank. Pooling of site-specific quintiles has been used previously^10^ and enabled analysis of the total cohort by applying a similar relative cut-off (i.e. top quintile), despite the biobanks representing a heterogenous material. Also, we did not have access to data on race/ethnicity and the results may therefore not be generalisable to other populations. In addition, the retrospective compilation of data may have implicated other biases affecting the results that we have not considered.

In conclusion, our results further support the hypothesis that relatively higher 25(OH)D concentrations may protect against the development of MS but not that the effect is stronger among young individuals.

## Supplemental Material

MSO892291 Supplemental Material - Supplemental material for High serum concentration of vitamin D may protect against multiple sclerosis Click here for additional data file. Supplemental material, MSO892291 Supplemental Material for High serum concentration of vitamin D may protect against multiple sclerosis by Martin Biström Lucia Alonso-Magdalena Oluf Andersen, Daniel Jons, Martin Gunnarsson Magnus Vrethem Johan Hultdin Peter Sundström in Multiple Sclerosis Journal – Experimental, Translational and Clinical

## References

[1] Munger KL Levin LI Hollis BW, et al Serum 25-hydroxyvitamin D levels and risk of multiple sclerosis. JAMA 2006; 296: 2832–2838. 17179460 10.1001/jama.296.23.2832

[2] Salzer J Hallmans G Nyström M, et al Vitamin D as a protective factor in multiple sclerosis. Neurology 2012; 79: 2140–2145. 23170011 10.1212/WNL.0b013e3182752ea8

[3] Munger KL, Hongell K, Åivo J, et al. 25-Hydroxyvitamin D deficiency and risk of MS among women in the Finnish Maternity Cohort. *Neurology* 2017; 89: 1578 LP – 1583. 10.1212/WNL.0000000000004489 PMC5634665 28904091

[4] Mokry LE Ross S Ahmad OS, et al Vitamin D and risk of multiple sclerosis: a Mendelian randomization study. PLOS Med 2015; 12: e1001866. 26305103 10.1371/journal.pmed.1001866 PMC4549308

[5] Rhead B Bäärnhielm M Gianfrancesco M, et al Mendelian randomization shows a causal effect of low vitamin D on multiple sclerosis risk. Neurol Genet 2016; 2: e97. 27652346 10.1212/NXG.0000000000000097 PMC5022843

[6] Simpson S van der Mei I. Vitamin D deficiency is an etiological factor for MS – commentary. Mult Scler 2019; 25: 641–643. 30499748 10.1177/1352458518815605

[7] Brink M Johansson L Nygren E, et al Vitamin D in individuals before onset of rheumatoid arthritis – relation to vitamin D binding protein and its associated genetic variants. BMC Rheumatol 2018; 2: 26. 30886976 10.1186/s41927-018-0033-8 PMC6390591

[8] Ramnemark A Norberg M Pettersson-Kymmer U, et al Adequate vitamin D levels in a Swedish population living above latitude 63°N: the 2009 Northern Sweden MONICA study. Int J Circumpolar Health 2015; 74: 27963. 10.3402/ijch.v74.27963 PMC4432023 28417824

[9] Snellman G Melhus H Gedeborg R, et al Determining vitamin D status: a comparison between commercially available assays. PLoS One 2010; 5: e11555. 20644628 10.1371/journal.pone.0011555 PMC2903481

[10] Marklund M Wu JHY Imamura F, et al Biomarkers of dietary omega-6 fatty acids and incident cardiovascular disease and mortality. Circulation 2019; 139: 2422–2436. 30971107 10.1161/CIRCULATIONAHA.118.038908 PMC6582360

