# Supplementary material for: High serum concentration of vitamin D may protect against multiple sclerosis
Source: Mult Scler J Exp Transl Clin. 2019 Dec 6;5(4):2055217319892291. doi: 10.1177/2055217319892291 (PMC6900627; doi:10.1177/2055217319892291)
Supplement: MSO892291 Supplemental Material - Supplemental material for High serum concentration of vitamin D may protect against multiple sclerosis [file MSO892291_Supplemental_Material.pdf]

Supplementary Figure 1. Flowchart of case ascertainment

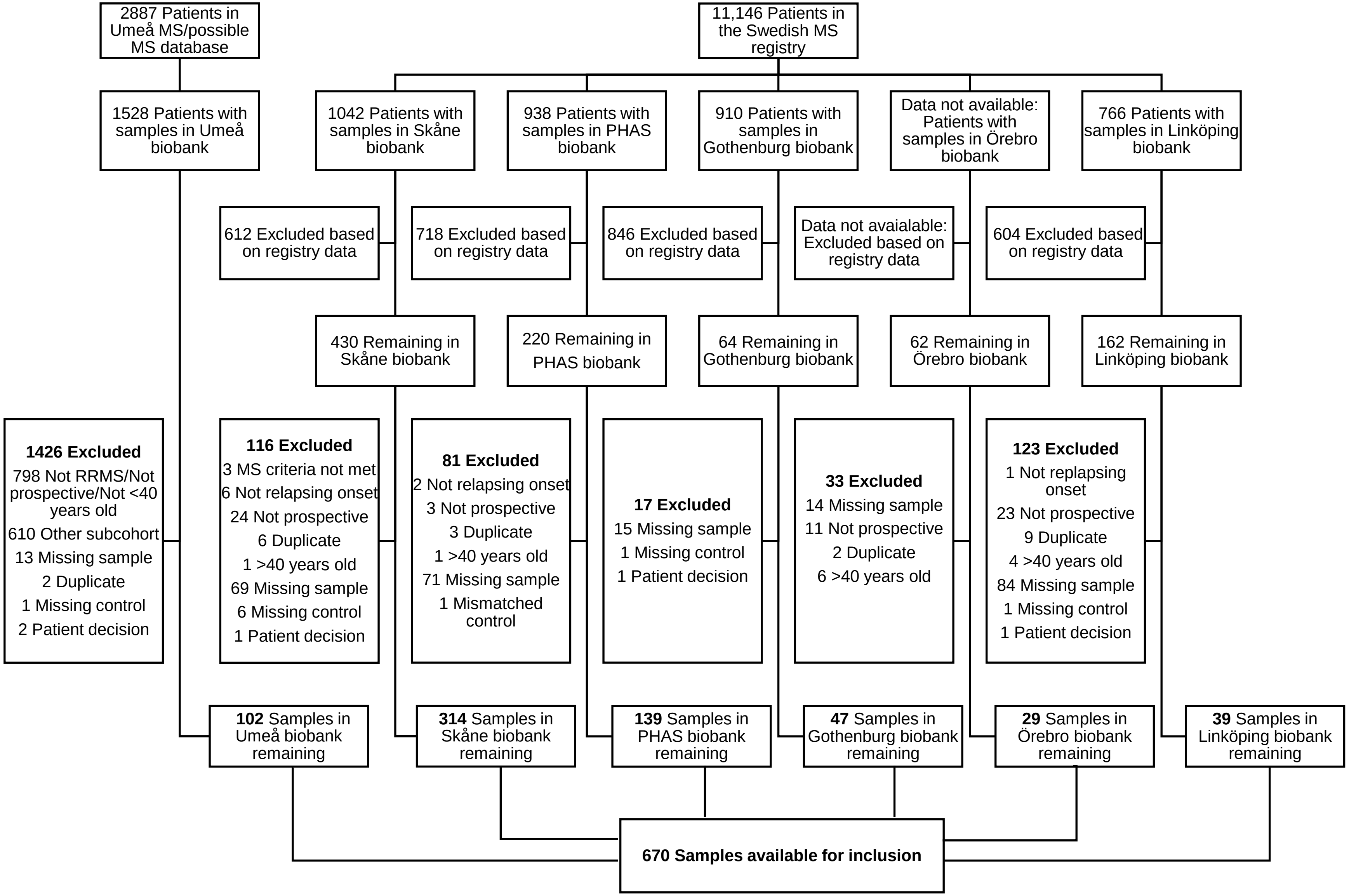

Legend Supplementary Figure 1: Samples collected before MS onset were identified through crosslinking between the Swedish MS registry or a local MS/possible MS database and six Swedish biobanks. In the next step, we excluded those not fulfilling study criteria based on registry data. When registry data were incomplete, medical records were used. These records, together with samples missing in freezers, the same individual having samples in multiple biobanks, and patients deciding not to participate in the study, resulted in further exclusions. A total of 670 individuals with prospectively collected samples were eligible to be included in the study but because of low sample volumes four more case-control sets were excluded. One additional set was excluded due to a disturbance in the chromatogram making the vitamin D asessment unreliable. The final analysis was therefore restricted to 665 cases and 665 matched controls. Inclusion criteria for the study was relapsing onset, <40 years of age and sample drawn before disease onset. PHAS = Public Health Agency of Sweden.
